# Adaptive Information Dissemination Control to Provide Diffdelay for the Internet of Things

**DOI:** 10.3390/s17010138

**Published:** 2017-01-12

**Authors:** Xiao Liu, Anfeng Liu, Changqin Huang

**Affiliations:** 1 School of Information Science and Engineering, Central South University, Changsha 410083, China; xiaoliu@csu.edu.cn; 2 School of Information Technology in Education, South China Normal University, Guangzhou 510631, China; cqhuang@zju.edu.cn; 3 Beijing Hetian Yuxiang Internet Technology Co., Ltd., Beijing 100036, China

**Keywords:** information dissemination, differentiated services, quality of service, communication cost

## Abstract

Applications running on the Internet of Things, such as the Wireless Sensor and Actuator Networks (WSANs) platform, generally have different quality of service (QoS) requirements. For urgent events, it is crucial that information be reported to the actuator quickly, and the communication cost is the second factor. However, for interesting events, communication costs, network lifetime and time all become important factors. In most situations, these different requirements cannot be satisfied simultaneously. In this paper, an adaptive communication control based on a differentiated delay (ACCDS) scheme is proposed to resolve this conflict. In an ACCDS, source nodes of events adaptively send various searching actuators routings (SARs) based on the degree of sensitivity to delay while maintaining the network lifetime. For a delay-sensitive event, the source node sends a large number of SARs to actuators to identify and inform the actuators in an extremely short time; thus, action can be taken quickly but at higher communication costs. For delay-insensitive events, the source node sends fewer SARs to reduce communication costs and improve network lifetime. Therefore, an ACCDS can meet the QoS requirements of different events using a differentiated delay framework. Theoretical analysis simulation results indicate that an ACCDS provides delay and communication costs and differentiated services; an ACCDS scheme can reduce the network delay by 11.111%–53.684% for a delay-sensitive event and reduce the communication costs by 5%–22.308% for interesting events, and reduce the network lifetime by about 28.713%.

## 1. Introduction

The Internet of Things (IoT) brings new trends and promising technologies to traditional Internet industries and society. Wireless Sensor and Actuator Networks (WSANs), as one of the pivotal core components of the IoT [1,2,3,4], are emerging as a promising platform that enables a wide range of applications in military, scientific, industrial and commercial applications. However, realizing the full potential of the Internet of Things requires solving serious technical and business challenges, such as the integration and management of big data, mobile communications and mobile computing, privacy and security issues. Wireless Sensor and Actuator Networks (WSANs) are composed of many wireless sensor nodes and relatively fewer actuator nodes because of the cost associated with the actuator nodes [1,2,3,4]. WSANs can monitor the environment by sensor nodes and send sensing data to corresponding actuator nodes. However, actuator nodes can judge, analyze and take action for an event [3,4]. Sensor nodes are tiny devices with limited sensing, energy, computation and wireless communication capabilities [5,6,7,8,9,10,11,12,13,14,15,16,17,18], thus, energy efficiency is a pivotal issue for network operations [19,20,21,22,23,24,25]. The wireless-enabled actuator nodes are resource-rich and equipped with better processing capabilities, higher transmission powers and longer battery life [1,2,3,12,26,27,28,29,30], or they can even be equipped with a robot that can move to a position where emergency event occurs, such as fires, which actuators can handle [4,27,28]. Compared with wireless sensor networks [5,9,14,18,23,25] that only passively monitor the environment, WSANs can quickly respond to events and take feedback action by actuators [1,2,3,4,27,29]. This feature of WSANs also renders them more widely used in many fields, such as environmental monitoring and civil and industrial areas [1,2,3,4,12,26,30]. WSANs combine with cloud computing [9], emerging mobile networks [10], cyber-physical systems [13], and crowd-sensing networks [19] to become an important basis for the application.

**Figure 1:**
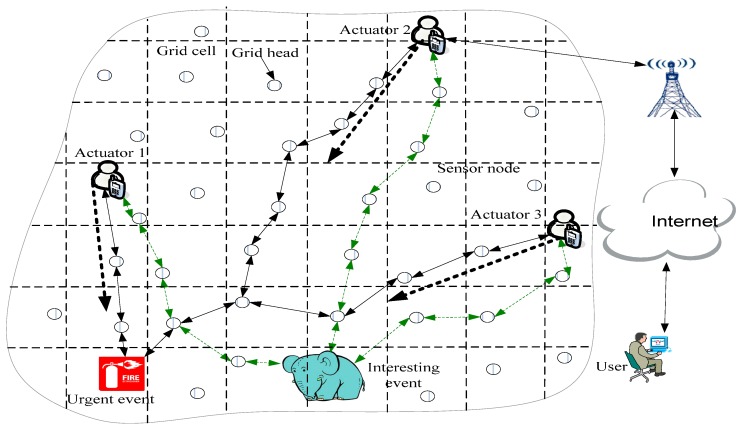
The architecture of WSANs.

Figure 1 presents the architecture of WSANs; the actuators in the files are used to sense a message and address an event. Sensor nodes are used to sense and transmit messages. In this field, the main method is to create routing between actuators and events to solve problems. However, there are some problems in these studies. Because the actuators and targets may move randomly at any time in the WSANs, how to design a route scheme in which an event message can be routed to an actuator quickly and efficiently is a challenge for WSANs. Some studies have addressed this issue, but there are still issues worthy of further study.

(1) Because actuators and targets may move randomly in the WSANs, the problem of how to send messages to actuators quickly and at low cost for urgent events cannot be easily resolved. It is critical to establish a route between actuators and events that enables the event to identify an actuator by some method. The flooding diffusion algorithm [8,18] is often used: actuators broadcast their location in WSANs, so all sensor nodes in the network can know the position of the actuators, and then the message can be sent to the actuators. Because so much energy can be consumed in routing diffusion algorithms, the routing diffusion method is not feasible in practice. In [8], Chi et al. proposed a tracking-assisted routing scheme to maintain the route between event and actuators without routing diffusion; however, this method only applies in cases in which a communication routing path from target to actuators can be established. For a sudden event such as a fire, the source node of the event (SNE) knows only the beginning position of the actuators. Therefore, using a tracking-assisted routing method can cause longer delays and higher energy consumption. Thus, a low-cost method that can be applied to different types of routes between event and actuators would be highly significant.
(2) Different events have different sensitivities to delay. Previous schemes, using the same method to solve this problem, rendered it difficult to meet the application requirements. It is crucial to send a message to actuators quickly for a delay-sensitive event; the issue of energy efficiency often gives way to the problem that the message should be sent to the actuator quickly [25,27,31,32,33,34]. For an interesting event, energy consumption and network lifetime are important indicators; however, delay is not the most important indicator. There is a conflict between energy effectiveness and delay. Therefore, a significant research topic is how to adapt differentiated delays to different requirements.

Based on the above analyses, this paper proposes an adaptive communication control based on a differentiated delay (ACCDS) scheme to resolve this conflict. Compared with previous schemes, the primary innovations of this paper are as follows:

(1) This paper proposes a differentiated delay framework for different delay-sensitive events in WSANs. To the best of our knowledge, the previous scheme used the same method to address events and cannot meet the various performance requirements of an event. The differentiated delay framework is proposed in this paper to resolve this conflict in WSANs. As a new type of solution to solve this problem, the differentiated delay framework is significant.
(2) The ACCDS method can create tradeoff optimization between communication costs and delay using the adaptive control method and meeting the requirements of the application. In the ACCDS scheme, an event sends various numbers of searching actuator routing (SAR) routes according to different degrees of sensitivity to delay. The scheme involves sending many SAR routes for an urgent event to reduce the delay in searching for an actuator but sending a small number of SAR routes for an interesting event to reduce energy consumption and improve network lifetime. In addition, actuators initiate limited diffusion routing based on their location and the route situation of an event, which can not only greatly reduce the huge energy consumption reported in previous studies using flooding routing but can also correct detour paths in previous events by the routing diffusion method of actuators. Thus, this scheme can reduce the time required to transmit a message from the event to the actuator and reduce network energy consumption. The ACCDS scheme has higher performance than the previous scheme and can be used in any event or scenario in WSANs.
(3) Our extensive theoretical analysis and simulation study demonstrate that the ACCDS scheme can reduce delay for a delay-sensitive event by 20%–57.143%, and communication for an interesting event can be reduced by 43.563%–52.379%.

The remainder of this paper is organized as follows: in Section 2, related works are reviewed. The system model and problem statement are described in Section 3. In Section 4, the details of the ACCDS scheme are presented. In Section 5, the performance of the ACCDS plan is analyzed in theory. Section 6 presents the experimental results and comparisons. Finally, we conclude in Section 7.

## 2. Related Work

The core problem of Wireless Sensor Actuator Networks (WSANs) is how to deploy the network most economically and monitor the network with energy efficiency and low latency by coordinating sensor nodes and actuators. Numerous issues must be studied with regard to WSANs [1,2,3,12,26,27,28,29,30]. Delay and communications costs are two pivotal issues for WSANs. Therefore, this paper focuses on the related research on delay and communication costs:

(1) Communication problems between sensor nodes and actuators. Selvaradjou et al. [14] noted that actuators have much greater energy and communication capabilities than sensor nodes do; thus, communication among actuators adopts communication methods with longer distances and higher throughput. Sensor nodes use communication manners with lower performance. Therefore, the system should adopt communication methods among actuators to the extent possible. However, for cost considerations, only a small number of actuators are deployed in WSANs; therefore, several partitions can be formed. The disadvantage of this plan is that the small number of actuators cannot satisfy the throughput of application requirements, and the large number of actuators is costly. Selvaradjou et al. [14] proposed the problem of how to establish effective routing issues among actuators by sensor nodes. Long et al. [29] extended the study of Selvaradjou et al., proposing a High Throughput Disjoint Multi-path (HTDM) routing scheme that allows actors to simultaneously forward data by multiple disjointed paths to achieve high throughput transmissions. Their theoretical analysis and experimental results indicate that the HTDM scheme can improve network throughput and is sensible. However, those studies focused on how to provide effective communication so that the network can communicate with others effectively and has fast or high throughput. For urgent events, the delay is not an important issue. The delay requirement should be considered in a new scheme [35,36].
(2) Studies on effective control actuator movement to improve data collection efficiency. In the above, the mobility of the actuator was not considered; the primary function of actuators is equivalent to sink nodes in WSNs. He et al. [26] proposed an Energy-Efficient Message Dissemination protocol (EMD) to collect data in delay-tolerant WSANs, grounded on the principle of “Carry-Disseminate-Store-and-Forward”. In EMD, the disseminated data can be stored in sensors in the movement process of the source actor. When the destination actuators pass the sensor nodes that store data, the stored data can be sent to destination actuators to achieve the purpose of data transmission. In these studies, the movement of actuators has been considered; however, the new location of the actuators cannot be properly stored in sensor nodes. It takes a long time to create a routing from source node to actuators, which reduces efficiency. For some delay-sensitive events, this method is low-efficiency. These plans do not consider delay. The efficiency in creating a route from actuators to event should be considered in the new scheme.
(3) Studies on the comprehensive consideration of event message delay. In some literature, actors walk randomly around the network before detecting events. When an event occurs, the distance from the actuators to the event is longer, the transmitted distance of energy consumption from event to actuator is higher, and the time when actuators move to the position of the event is longer. Although this scheme considers the delay, a large event detection delay and higher energy consumption remain. Dong et al. [4] proposed an approach called RENDEZVOUS to accelerate the actor’s event-detecting process while maintaining minimal energy consumption of the sensor nodes. To achieve the above purpose, the actuators are controlled to move close to the event using Reinforcement Learning techniques. ORACLE is another approach to reducing the delay in actuators’ processing an event by predicting its location [30]. First, the proposal includes an event prediction scheme to predict an event by utilizing the maximum likelihood estimation. Based on the predicted results, the actuators are moved to the annex of the event in advance; thus, when the event occurs, the delay in addressing the event can be greatly reduced. Because the event and the actuators may move simultaneously, how to maintain effective communication is another research issue.

**Figure 2:**
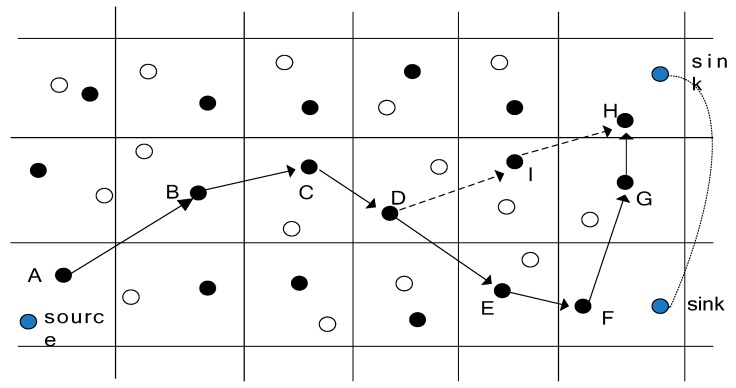
The description of TRENS.

The GMDQP scheme builds the grid structure from the sink side. As shown in Figure 2, when the sink queries a target for the first time, it selects a node as the primary data node for initiating the grid constriction. The primary data node calculates the location of four adjacent cross points (DEP) on the grid. Then it floods the sink’s query message in order to select four new data examination nodes near the DEP. Each DEP repeats this action to elect adjacent new DENs, until a grid structure is built. When the sink moves, a new agent can be selected. The shortage of the GMDQP scheme is that the position of the mobile agent must be kept all the times, which can cause higher energy consumption.

The CODE scheme selects a coordinator to transmit the data in each grid cell. As the source senses the message from the environment, it floods a message containing his location to all coordinators in the network, then the sink builds a routing path toward the source by forwarding a data query message. The sensed message by source are transmitted to the sink along this building routing. When the sink moves to another places, it selects a new agent, which is responsible for rebuilding the new routing path. These rebuilding operations entail explicit overheads. When the sink moves quickly, the new agent must be selected on a frequent basis, thus resulting in significant overheads.

Chi et al. [28] proposed a tracking-assisted routing scheme called TRENS that can effectively create a route from event to actuator in the case of a moving target. The scheme initializes the grid structure with the node’s geographic location, it selects a grid head in each grid cell to transmit data. When the source senses data from the surrounding, it floods a message containing its location to all of the grid heads before reporting the data, then the sink builds a routing to the source node by transmit a query message. The message sensed by sensor nodes can be transmitted to the sink. But when the sink node moves, as showed in the Figure 2, the shortcutting packet can be sent from the sink to the node, then the shortcutting path can be built. For example, if the sink moves from F to H, it can be seen that the hop counts in the routing path A-B-C-D-E-F is 5, and the hop counts in the routing path F-G-H is 2, The total hop number from source node to the sink is 7. In the TRENS scheme, when the sink moves to H, the hop counts can be calculated according to the distance and the transmission radius. If the calculated hop counts is smaller than the real hop counts, the shortcutting routing path can be built. It can be seen that the hop counts in the routing path D-I-H is 2, and the hop counts in the routing path A-B-C-D is 3. The hop counts in the routing path A-B-C-D-I-H is smaller than the hop counts in the routing path A-B-C-D-E-F-G-H, thus, the routing path must be shorten. The shortest routing path is A-B-C-D-I-H.

The shortcoming of this scheme is that when the source node floods the location message to the whole network, more energy can be consumed. When a sink moves to another location, the shortest routing can be rebuilt, but this will require extra transmission delays. If the data is transmitted to the sink in the process of building the routing, data can be lost. In these studies, energy consumption and event delays are important issues. The proposed scheme should simultaneously address the delays and the energy consumption to achieve an optimal value.

## 3. The System Model

### 3.1. The Network Model

(1) According to several studies [2,3,12,29], a large number of sensor nodes and several actuators are randomly deployed in the WSAN. The maximum transmission ranges of actuators and sensor nodes are represented by $r_{a}$ and $r_{s}$, respectively. The neighboring distance is the maximal reachable distance with the transmission power for neighboring sensors/actuators. For a given sensor/actuator, the sensors/actuators within its neighboring distance are its “neighboring sensors/actuators” or “neighbors”. Each sensor can receive information within the transmission radius and send information to actuators/sensors. We primarily focus on the protocol design in the routing layer. The data transmission in the MAC layer and link layer is simplified. The bandwidth, traffic load and network conditions, among other factors, have been simplified. This paper addresses how to reduce the delay, thus, the bandwidth, transfer delay, and losses are simplified, and the simplification is reasonable. Thus, this paper focuses on the delay.
(2) Each node/actuator is aware of its location via the receiver of a global positioning system (GPS) or some other location-estimation device [3,28]. All sensor nodes receive the location of all actuators after the initial phase, when the event occurs. The message regarding this event is sent to actuators according to the stored location message. If actuators move to another location, a message regarding the new location is sent along horizontal and vertical directions. When the message regarding the event is transmitted to the sensor node that stores the actuators’ new location, the message is sent to actuators along this tracking path. All sensor nodes have limited energy whereas the actuators have unlimited energy.

Figure 1 illustrates the WSANs’ architecture. First, numerous wireless sensor nodes and a small number of actuators are pre-deployed in the monitored area, these sensor nodes can perceive and monitor anywhere in the monitored area. The wireless sensor is deployed and cannot be moved although the actuator and the monitored target can move [1,2,3,4,12,26]. The actuator is connected to the Internet wirelessly; thus, the user can know the situation of the monitored area [27]. The objects monitored by WSANs may be moveable targets such as wild animals or enemy vehicles [28], or the targets may be stationary, such as the temperature or humidity of a location or a fire. In order to monitoring the temperature or humidity of the live environment, sensor nodes in this filed is statistic to monitoring the environment, it is used to ensure the safety of the environment. All detection targets are defined as events in this paper. Events can be divided into high-urgent events, urgent events, or low-urgent events according to the emergency level of the event. To address the event, actuators must sometimes move to the location of the event.

Although sensor nodes in the network cannot be moved, the event (target) is mobile. The model is very suitable for environmental monitoring. For example, sensors monitoring fire events, which are high-urgency, send the monitored message to the actuator as soon as possible; the actuator may be a firefighter using a WSAN to monitor forest fires. High-urgent events are quite sensitive to delays. If event information cannot be transmitted to actuators quickly, serious losses can occur. The event that monitors wild animals in a wildlife park is a low-urgency event, that is, not sensitive to delay. In this situation, the position of wild animals may be transmitted to tourists (actuators) who are in touring cars. In the following, the urgent event is sensitive to delay; however, the interesting event is delay-insensitive.

### 3.2. Event Model

(1) All sensor nodes are stationary, the actuator is movable, and the node churn is not considered. When detecting an event, the sensor node that receives the strongest signal becomes the source, and the sensing data must be routed to actuators via the wireless channel.
(2) Events can be divided into a variety of situations. Events can also be divided into two types depending on whether the actuator is involved in the event: (a) an event can be resolved only when actuators move to the position of the event source, such as a fire; and (b) an event can be resolved without an actuator, for example, when the sensor node discerns an interesting animal event, the event message is sent to actuators to inform the actuators (tourists) of the current location and status of the animals. An event can be divided into k levels depending on the degree of sensitivity to delay: Level 1 is the most sensitive to delay, and higher level numbers indicate less sensitivity to delay. The maximum allowable delay of k levels is set at $D_{k}$; thus, $D_{1}\leq D_{2}\leq \ldots \leq D_{K}$. When the event message regarding an urgent event is transmitted to the actuators, the event sends a large number of actuators searching actuators routing. However, in practice, fire is Level 1, and the degree of sensitivity to delay is high, resulting in disastrous consequences without timely treatment. For an interesting event, however, the degree of sensitivity to delay is small, resulting in less damage without real-time updates of information. An event is produced by monitoring the destination. Because the monitoring destination may be stationary or moveable, an event may be stationary or moveable.

### 3.3. Energy Consumption Model and Related Definitions

The energy consumption model adopted in this paper is consistent with the model in references [25,29] and is an improved typical energy model. The original energy model, called “first order radio model”, can only be used in a system where all sensors are sensing the environment at a fixed rate and thus always have data to send to the end-user. In order to avoid the limitation, the energy model is enhanced. In this paper, improved energy consumption is adopted.

The energy consumption for transmission $E_{t}$ is denoted in Equation (1), and energy consumption $E_{r}$ for receiving, is denoted in Equation (2). $E_{elec}$ represents transmitting circuit loss. Both the free space ($d^{2}$ power loss) and the multi-path fading ($d^{4}$ power loss) channel models are used in the model, depending on the distance between the transmitter and the receiver. $\varepsilon_{fs}$ and $\varepsilon_{amp}$ are, respectively, the energy required by power amplification in the two models; l denotes the data bits. The above parameter settings can be observed in [25,29]. The above parameter settings are presented in Table 1, as adopted by [25,29].

(1) $$\{\begin{array}{c} E_{t}=lE_{elec}+l\varepsilon_{fs}d^{2},\;if\;d<d_{0}\\ E_{t}=lE_{elec}+l\varepsilon_{amp}d^{4},\;if\;d>d_{0} \end{array}$$

(2) $$E_{r}=lE_{elec}$$

### 3.4. Problem Statement

The aim in this paper is to reduce the transmission delay in the process of data transmission. This paper mainly focus on the protocol design in routing layer.

**Definition** **1.** Delay is defined as the time from the production of an event to the actuator’s receiving the event message. In the process of transmitting data, the hop count for creating routing from event to actuators is the primary factor affecting delay; though the data transmission in MAC layer and link layer are streamlined, the MAC, queuing and propagation are considered. The paper aims to reduce the transmission delay while retaining higher network lifetime. Though this paper mainly concerns the transmission delay, it has great significate for the application requirements. For example, sensors monitoring fire events, which are high-urgent, send the monitored message to the actuator as soon as possible; the actuator may be a firefighter using a WSANs to monitor forest fires.

**Definition** **2.** *Path benefit coefficient* δ *is defined as the ratio of the length of the routing path from event to actuator to linear distance between actuators and the event. Clearly,* δ *= 1 indicates the shortest path from event to actuator. The larger the* δ *is, the longer the length of the path from event to actuator and the poorer the performance.*

The primary focus of this paper is to design a new, effective routing scheme from event to actuators. The scheme can adopt differentiated delays for different events. However, shorter delays and lower communication costs suggest a better scheme. Different events have different performance requirements: for urgent events, the delay should be as short as possible; for an interesting event, the cost for routing the message to actuators should be lower. The goal of the adaptive communication-control-based differentiated delay (ACCDS) scheme can be categorized as follows:

(1) Scheme can meet different delay requirements for different events.
     The delay can be evaluated as the requirement time for transmitting data from event to actuator. Thus, the first target scheme is to minimize the delay $D_{i,k}$ of any event i belonging to k level on the premise that the delay $D_{i,k}$ is less than the maximum allowable delay $D_{k}$. That is, different events have different delay requirements. The delays for transmitting data for different levels should meet the delay requirements of different levels and minimize delay:
  (3) $$\text{min}\left(D_{i,k}\right)\;s.t\;D_{i,k}<D_{k}$$
(2) Minimizing the cost of communications. The cost of communications is defined as the communication costs when events communicate with actuators. The cost of communications can comprise the following: (a) the event reports the requirement communications volume to actuators when the event occurs. The communications volume can be measured by the length of the path from event to actuators, denoted as $c_{1}$; (b) the cost of diffusing the routing information of actuators considers that the length of the diffusing packet of the actuator is equal to the length of the message. Thus, the cost can be measured by the hop number of diffusing information, denoted as $c_{2}$; (c) the communication costs for searching for actuators of an event is denoted as $c_{3}$; (d) the cost of sending a message from the actuators to the event is denoted as $c_{4}$; (e) the movement costs when actuators move to the location of an event, which can be displayed as the movement hop number of actuators, is denoted as C. In this paper, we mainly focus on the protocol design in routing layer, the main concern in this paper is how to reduce the routing length. The formula of minimization of the communications costs can be expressed as follows:
  (4) $$\text{min}\left(ℭ\right)=\text{min}\left(c_{1}+c_{2}+c_{3}+c_{4}\right)+\text{min}\left(C\right)$$
     Generally, tradeoff optimization exists in the performance indexes above. Less delay C leads to greater communications costs. In summary, the optimization purpose of the scheme in this paper is:
  (5) $$\{\begin{array}{c} \text{min}\left(D_{i,k}\right)\;for\;each\;event\;i\in k\;level\\ \text{min}\left(ℭ\right)=\text{min}\left(c_{1}+c_{2}+c_{3}+c_{4}\right)+\text{min}\left(C\right)\\ s.t\;D_{i,k}<D_{k} \end{array}$$

## 4. Design of the ACCDS Scheme

If the sink moves quickly in the TRENS scheme, it performs the rebuilding routing process on a frequent basis, which results in significant overhead, but when the source sends data while rebuilding a shorter routing path, the data may be lost. In the proposed scheme, in order to bridge the shortcoming of larger transmission delays, three aspects are used to improve the performance: (1) if actuators move to another location, the message regarding the new location is sent in horizontal and vertical directions. When the message regarding the event is transmitted to sensor nodes that store the actuators’ new location, the message will be sent to actuators along this tracking path; (2) if the event is urgent, the larger number of searching actuators routing (SAR) can be initiated by source nodes in different directions, thus, the message can be sent to the actuators in a timely way; (3) if the routing path is too longer, when actuators receive the message from source nodes, actuators initiate a shortcutting II route directly to the event. Compared to the TRENS scheme, the transmission delay can be reduced, and the data can be transmitted to the actuators successfully.

### 4.1. The Overview of the ACCDS Scheme

The initial phase of the ACCDS scheme is displayed in Section 4.2. The initial phase is consistent with reference [28]. When an urgent event occurs, that event routes searching actuators to all actuators. The method for searching for actuators is presented in Section 4.5. Some actuators, however, move to another location to report the new location to the sensor nodes. If the distance from the original location to the new location is short, the method for tracking the actuators is displayed in Section 4.3; if the distance is long, the method for tracking actuators and events is displayed in Section 4.4. The information regarding an urgent event is sent to all actuators, using the tracking method for actuators. The actuators that receive information will move to the location of the urgent event to resolve the problem and remain until the urgent event is resolved.

**Figure 3:**
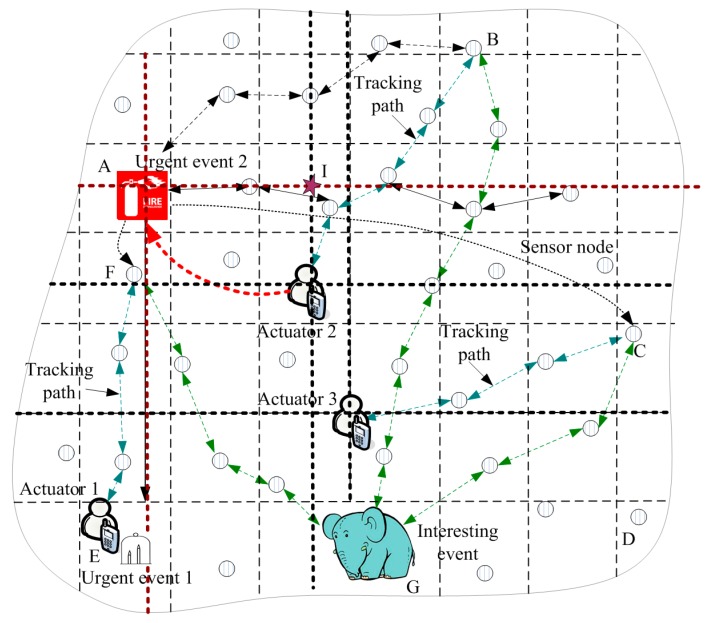
The status of WSANs after generating Urgent Event 1.

In Figure 1, the process for resolving an event is presented. When an urgent event occurs, the event sends searching actuators routing (SAR) to actuators. When the actuators (such as Actuator 2) move to another location, $G_{i}$, in which the actuators are located, sends a packet that contains location information to GH along the right and left of the horizontal direction. The sensor nodes in this routing path store the location of the actuators, represented by the dotted line around Actuator 2 in Figure 3. When SAR is transmitted to one sensor node (such as I in Figure 3) that stores location information regarding the actuator, the SAR will know the new location of the actuators, and then SAR is transmitted to the location of the actuator. In Figure 1, when actuators receive a message from an urgent event, $A_{1}$ first moves to the location of Urgent Event 1 (denoted as $L\left(e_{1}\right)$) to extinguish the fire, and $A_{2}$ and $A_{3}$ also move to $F\left(e_{1}\right)$ after receiving a message from $e_{1}$. $A_{1}$ arrives at $F\left(e_{1}\right)$ sooner than $A_{2}$ and $A_{3}$ arrive at $F\left(e_{1}\right)$; thus, $A_{2}$ and $A_{3}$ stop moving because the message from $e_{1}$ was not received. The status of WSANs at this time is presented in Figure 3.

### 4.2. Preliminary Phase of ACCDS Scheme

Initializing the ACCDS scheme is consistent with the scheme in Ref. [28]. WSAN is divided into a number of grid cells (see Figure 1), and each grid cell is assigned a unique identifier. The grid size is ℬ×ℬ and $ℬ=r_{s}/2\sqrt{2}$, allowing the cell to communicate directly with its eight adjacent grid cells [28]. A special node is selected in each cell as the grid head, which is responsible for receiving and forwarding packets. Sensor nodes in a cell except the GH are called grid members (GM), which are responsible for sensing data (or an event) and forwarding the information to the GH. The event message is routed to actuators only through GHs [28].

The ACCDS scheme adopts a flooding routing method to establish the initial path; the primary point is that the hop count is set to 0 by each actuator, and the hop count of the sensor nodes to all actuators is set at infinite. Then each actuator, such as $A_{i}$, broadcasts the information that the hop count to $A_{i}$ is 0; the hop count to $A_{i}$ can be added to when a node receives the broadcast information. The original hop count is then compared to $A_{i}$ and stored in the node. If the original hop count to $A_{i}$ is smaller than the broadcast hop nu count, the stored hop count can be replaced by the broadcast hop count. Then the updated hop count to $A_{i}$ can be broadcast after a short time. The process continues for some time or until the hop count of all the networks cannot be updated. Thus, each node can determine the minimum hop count to each actuator. If the number of actuators in WSAN is N, the routing table of each sensor node routing table (RT) includes N tuples. Each tuple contains information (destination, next, hops, time) in which the destination is the ID of the actuator. “Next” is the downstream ID of the GH; “hops” is the hops to the destination actuator, and “time” is the latest time of this tuple to be updated.

When an event occurs, the source node reports sensing data as a message to the location GH. GH D seeks its RT and obtains the tuple, which contains the destination ID of the destination actuator. Then, the message is sent to the referred next GH. The GH that receives the message repeats the above process, which can send messages of event routes to actuators that the GH wants to route (see Figure 1).

### 4.3. Tracking Actuators and Events in the ACCDS Scheme

Because the the flooding routing consumes huge amounts of energy, the ACCDS scheme is used only once in the initialization phase. However, actuators and events may move from time to time, which will lead to the failure of the above routing method. Thus, when the movement distance of actuators or events is small, the tracking path can be established in the ACCDS scheme by adopting a tracking target scheme [28] to ensure that the route between event and actuators functions properly. The method of tracking actuators is as follows: when the GH $G_{1}$ of a cell located by actuator $A_{i}$ monitors the situation that the actuator is running into an adjacent GH $G_{2}$ of a cell, $G_{1}$ informs the $G_{2}$ monitoring actuator and updates the corresponding tuple of the RT that arrived at $A_{i}$. Although the other nodes cannot know the movement situation of an actuator, an event can nevertheless send a message to $G_{1}$ along the original routing method. Then the message is routed to $G_{2}$. The examples are presented on the route $B\to A_{2}$, $F\to A_{1}$, $C\to A_{3}$ in Figure 3.

When an event is moved to a new cell, the GH of the new cell can route to the next GH according to their own routing table. However, when an actuator initiates shortcutting II to an event, if the event moves to another place at that time, the message launched by the shortcutting II routing cannot be sent to the event. Thus, the system requires the path for the tracking event. This method is identical to the method for tracking actuators. When the GH $G_{1}$ of a cell located by event $e_{1}$ monitors that $e_{1}$ is passing into the cell located by a neighboring GH $G_{2}$, then $G_{1}$ informs $G_{2}$ monitoring $e_{1}$ and establishes routing tuples to $e_{1}$ (event-ID, next, time), in which the “event-ID” is the ID of the event and “next” is the downstream ID of the GH for routing the message to the destination event.

### 4.4. Dissemination Actuators’ Location Scheme in the ACCDS Scheme

Although the above method can establish a route between an event and the actuators, the actuators and the event only know the initial location. When actuators and event move to another place, the route is nevertheless sent to the original position, and then the message can be transmitted along the tracking path to the destination. Thus, as the distance from the event to the actuators increases, the curved delivery path becomes longer (see Figure 3), resulting in a greater delay and increased energy consumption. In the ACCDS scheme, a bounded routing path to disseminate the location (BRPDL) approach (Algorithm 1) is proposed that can establish effective routing from the actuators to the event and overcome the shortcoming of the huge energy consumption of flooding routing. In the ACCDS scheme, the BRPDL approach can be executed when the location information must be diffused. The process of the BRPDL approach is that an actuator such as $A_{i}$ forms a message packet that contains the tuple (actuator-ID, location, time, type), in which “type” represents type 1 routing diffusion. Then the packet can be sent to the GH $G_{i}$ of the cell located by the actuators. $G_{i}$ sends the packet to the GH along the right and left of the horizontal direction. The ID and location of the actuator can be extracted when each GH receives a packet. The tuple (actuator-ID, location, time) is formed and is stored in the location. The packet continues moving forward until being routed to the network border. Simultaneously, the packet can be sent to the network border by $G_{i}$ using a similar method along the vertical direction. Thus, two crossing routes at the intersection of the actuator can be formed, and all GH nodes in those 2 paths can store the ID and location of $A_{i}$.
**Algorithm 1:**
A bounded routing path to disseminate the location (BRPDL) of the actuators’ approach1:  **For** each actuator $A_{i}$ that needs to disseminate its location **Do**2:   $A_{i}$ form a packet P that includes tuples such as     ($A_{i}.$ID, $A_{i}$.location, current-time, 1);3:   $A_{i}$ send packet P to the GH $G_{i}$ of location cell;4:   **Do while**
$G_{i}$ is not at the cell of network boundary5:     $G_{i}$ send packet P to the right GH $G_{k}$;6:     $G_{k}$ store the tuple ($A_{i}.$ID, $A_{i}$.location, current-time);7:     $G_{i}=G_{k}$;8:   **End Do**;9:   Let $G_{i}$ be the GH of the cell, which $A_{i}$ locate;10:   $G_{i}$ send packet P to the left GH $G_{a}$;11:   $G_{a}$ route P to the left GH until network boundary, and store tuple ($A_{i}.$ID, $A_{i}$.location, current-time) in each GH of routing path;12:   $G_{i}$ send packet P to the up GH $G_{b}$;   $G_{b}$ route P to the up GH until network boundary, and store tuple ($A_{i}.$ID, $A_{i}$.location, current-time) in each GH of routing path;13:   $G_{i}$ send packet P to the down GH $G_{c}$;   $G_{c}$ route P to the up GH until network boundary, and store tuple ($A_{i}.$ID, $A_{i}$.location, current-time) in each GH of routing path;14:  **End For**

In the ACCDS scheme, the actuator uses another location dissemination approach. If the length of the path from $e_{i}$ to $A_{i}$ is longer than a predetermined threshold when actuator $A_{i}$ receives a message from $e_{i}$, $A_{i}$ initiates a shortcutting II route directly to $e_{i}$. The message, whose contents contain ID, $F\left(e_{1}\right)$, location and route type 2 (2, stand shortcutting II route), can be sent by $A_{i}$ to GH $G_{i}$ located by the cell. The route type can be obtained by reading the route type of the message when $G_{i}$ receives the message. Then the message can be sent to GH $G_{a}$ nearest $e_{i}$. By adopting a geographic routing approach, $G_{a}$ first produces the route tuple to $A_{i}$ (actuator-ID, event-ID, next, time), in which “next” refers to the source GH of the message. This process is repeated until arrival at the GH $G_{1}$ located by $e_{1}$. After that, the message of $e_{1}$ can be sent to $A_{i}$ by $G_{1}$ along the new route table by reverse shortcutting II.

### 4.5. Creating Routing from Event to Actuator in the ACCDS Scheme

In the ACCDS scheme, there are two situations for creating a routing path from an event to actuators. These situations are discussed below. The GH of event $e_{1}$ is denoted as $G_{e,1}$; the destination actuator is $A_{i}$.

**Figure 4:**
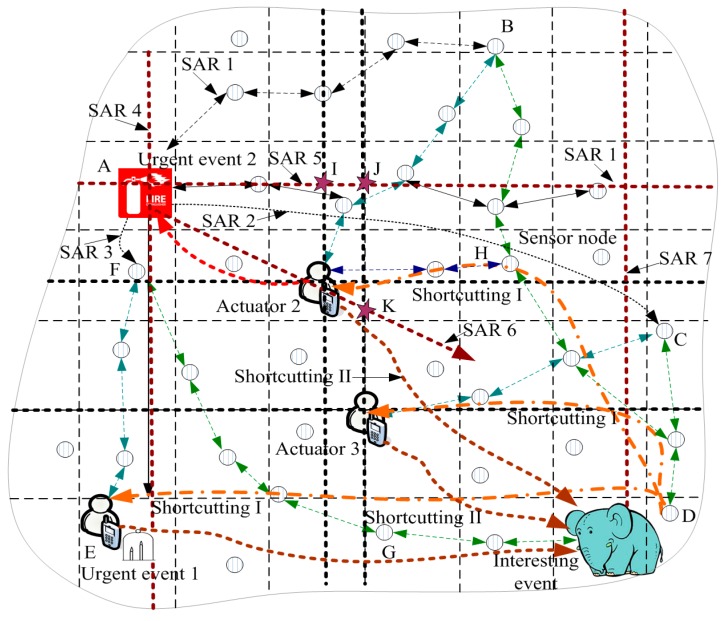
The status of WSANs after generating Urgent Event 2.

**Figure 5:**
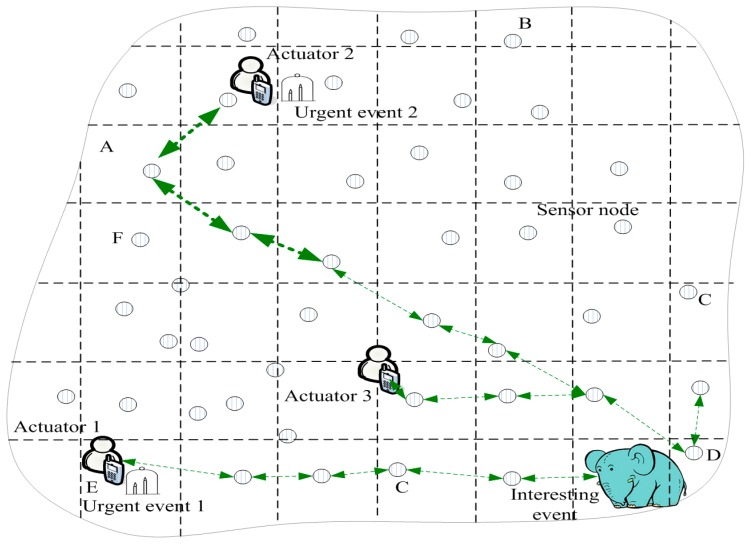
The result of adjusting the routing path in WSANs.

(1) Established active and updated mode. The ACCDS scheme adopts a differentiated delay approach: when event $e_{1}$ occurs, $G_{e,1}$ sends X searching actuators routing (SAR) routings according to the sensitivity to delay of event $e_{1}$. Those X SAR routings contain the stored location information of the actuators in $G_{e,1}$. For example, in Figure 4, $G_{e,2}$ stored the location information of $A_{1}$, $A_{2}$, and $A_{3}$; thus, the three routings of SAR 1, i.e., SAR 1, SAR 2, and SAR3, with destinations can be sent. However, the three routings of SAR 4, SAR 5, and SAR 6 are the routings for searching actuators without a destination. For an interesting event, the routings to $A_{1}$, $A_{2}$, and $A_{3}$ are established (regarded as a special SAR); only the searching routing, SAR 7, must be added. In an attempt to identify a better route, those X routings should be evenly distributed over the network to obtain the best search results. The message in SAR routing should contain the event-ID, event-location, event-time, and multiple tuples. Each tuple for an actuator is similar to actuator-ID, actuator-location, latest-time, in which “event-time, latest-time” represent the time at which the event occurred and the location updated time of the actuator, respectively.
     SAR sends a query regarding this problem, asking whether the location information of the actuators is stored in a node that the SAR passes during the routing process of SAR. If the stored location message of the actuators is more current than the transmitted location message (such as $A_{i}$), then geographic routing can be initiated from the forwarding node to $A_{i}$. A new geographic routing can be initiated when encountering new location information of the actuators. The original SAR continues moving forward until routed to the network edge or the SAR arrives at the actuators. For example, in Figure 4, SAR 5, initiated by $e_{2}$, meets the location disseminated routing path of actuators $A_{2}$ and $A_{3}$ at the intersection of I and J, resulting in the geographic routing from I, J to $A_{2}$, $A_{3}$ being launched. Obviously, compared with the length of the routing from the initial position to the actuator, the length of the path is greatly reduced. More SAR renders it more likely that the best routing will be identified; for example, the identified routing by SAR 6 initiated by $e_{2}$ is nearly optimal.
     Actuators can receive one of two types of messages from an event. The first type is an event that must be processed when the actuator moves to the event location. Actuators move to L(e) at this time. Another type is an event in which the actuator does not move. Actuators send a message directly to $G_{e,1}$. The routing from $G_{e,1}$ to the actuators can be established (for example, in Figure 5, the routing is shortcutting II routing).
(2) The automatically updated manner of processing message routing. If the $G_{e,i}$ message route of the event moves along the established routing path, which is from the event to $A_{i}$, after a while, $G_{e,i}$ identifies the updated location information of $A_{i}$ in the routing path. The routing from $G_{e,i}$ to $A_{i}$ can be changed. In Figure 4, an interesting event is routed along the routing path of $D\to B\to A_{2}$. If the location dissemination of the routing of $A_{2}$ has been completed, the interesting event can intersect the location disseminated routing path at point H. Thus, the routing path can be updated as $D\to H\to A_{2}$. The routing path from interesting event to $A_{1}$, $A_{3}$ can also be updated in the same fashion (see Figure 4). Creating the event to the actuators’ routing scheme can be described as Algorithm 2.

**Algorithm 2:**
Creating the event to the actuators’ routing scheme**Type 1:**
For event launch to create a routing path to the actuator;1:  The event $e_{i}$ computes the number of SAR as X2:  $e_{i}$ sends X and tuple (*event-ID, event-location, event-time*) denoted as $T_{e_{i}}$ to the GH $G_{i}$ of the cell that $e_{i}$ locates;3:  $G_{i}$ forms a packet P that includes $T_{e_{i}}$ and *actuator-ID*, *actuator-location*, and *latest-time* for each actuator;4:  $G_{i}$ launches a SAR to each actuator with packet P;5:  **If** the number of actuators N < X6:   $G_{a}$ launches X-N straight lines SAR, which are evenly distributed;7:  **End if**8:  **For** each GH $G_{a}$ in the SAR **Do**;9:   **If**
$G_{a}$ stores updated location information of actuators;10:    $G_{a}$ launches a SAR to each up-to-date actuator;11:   **End if;**12:  **End For;**13:  **For** each actuator $A_{i}$ that receives the packet P
**Do**14:  **If**
$e_{i}$ needs $A_{i}$ to move on15:   $A_{i}$ moves to $e_{i}$;16:  **Else**17:   $A_{i}$ launches a shortcutting II routing to $e_{i}$;18:  **End if**  **Type 2:** For message on the routing path to actuator;19:  **If**
$G_{b}$, **which** forwards the message, has updated location information of actuator $A_{i}$;20:   The routing path update routing path to the new location of $A_{i}$;21:  **End if;**

## 5. Performance Analysis and Optimization

### 5.1. The ACCDS Scheme Optimization

In the ACCDS scheme discussed above, the following four important parameters were not included: (1) the time for launching the location disseminate routing of the actuator; (2) the time for initiating shortcutting II routing of the actuator; (3) the time for sponsoring the SAR of an event, and (4) the value of the number of SA. The optimized value of those parameters is presented in this section.

(1) The time for launching location disseminate routing of an actuator depends on the movement of the actuators. Clearly, if the movement path of an actuator is long, the location information must be diffused over a longer distance to obtain the location of the actuator. Thus, the information can be diffused by the event along the last diffusion routing, then along the tracking path to the actuator. Thus, the longer the moving distance is, the longer the tracking path is, increasing the delay and costs. Conversely, if the actuator has not been moved, it is not necessary to diffuse location information because the stored information in sensor nodes is the current status. The cost-based method is used in this paper; that is, when the obtained revenue for diffusing location information is greater than its cost, the BRPDL algorithm can be used. The required cost of the BRPDL algorithm, the energy consumption process, is as follows: The network is L×L areas, and the edge length of each cell is $ℬ=r_{s}/2\sqrt{2}$. The number of the GH is $ℳ={\left(L/ℬ\right)}^{2}$. The diffusion routing is launched in the GH. The number of the GH in a diffusion routing is $\sqrt{ℳ}$, and the number for launching location diffusion is 2. The energy consumption for the position diffusion algorithm is Equation (6), where $\epsilon_{t}$, $\epsilon_{r}$ are energy consumption for receiving and sending a packet:
  (6) $$c_{2}=2\sqrt{ℳ}\left(\epsilon_{t}+\epsilon_{r}\right)$$
     Because the number of events reported by $A_{i}$ is 𝓎, the movement distance from the last diffusion location to the current location is ${𝒽}_{new}$ hops. The current position of $A_{i}$ is ($X_{A_{i}},Y_{A_{i}}$), and the current position of event $e_{i}$ is ($X_{e_{i}},Y_{e_{i}}$). The hop number from event $e_{i}$ to the last diffusion position is ${𝒽}_{old}$. The straight hop number from event $e_{i}$ to $A_{i}$ is ${𝒽}_{dir}$, and the average number of sending packet of $e_{i}$ after one update, is ${ℊ}_{i}$. Thus, the payoff of $A_{i}$ for event $e_{i}$ after the last position is:
  (7) $$S_{i,i}={ℊ}_{i}\left({𝒽}_{old}+{𝒽}_{new}-{𝒽}_{dir}\right)\left(\epsilon_{t}+\epsilon_{r}\right)$$
     The total payoff of $A_{i}$ after the location is updated is thus:
  (8) $$S_{i}=\sum_{k=1}^{𝓎}S_{i,k}$$
     After $A_{i}$ moves to a new location, when $S_{i}$ > $c_{2}$, the position diffusion algorithm can be initiated:
(2) The time of initiating shortcutting II route of the actuator. The cost is $c_{4}={𝒽}_{dir}\left(\epsilon_{t}+\epsilon_{r}\right)$ when $A_{i}$ launches a shortcutting II routing for event $e_{k}$, and the payoff is $U_{i,k}=h_{k}\left({𝒽}_{old}+{𝒽}_{new}-{𝒽}_{dir}\right)\left(\epsilon_{t}+\epsilon_{r}\right)$, where $h_{k}$ is the number of the sending packet after the shortcutting II route is updated once. Obviously, when $U_{i,k}$ > $c_{4}$, $A_{i}$ launches a shortcutting II route for event $e_{k}$.
(3) The time of sponsoring a SAR of an event. If an event has just occurred and the route from source to actuators cannot be established, SAR can be initiated. For an event for which the route to the actuator was previously established, SAR can be initiated by the event if the sensitivity to delay of the event is increased.
(4) The number of SARs. In the ACCDS scheme, the number of SARs is adaptively adjusted based on the urgency of the event. First, SAR sends the route to the actuator to ensure that the actuator can be located. Second, initiating SAR can be initiated in vertical and horizontal directions. It can be ensured that the route must intersect with the diffusion route of the actuators, allowing the actuators to be located quickly. However, for an event that is extremely sensitive to delay, more SARs can be initiated from many directions. The minimum number of SARs is the number of actuators. For an interesting event, adding a SAR in the horizontal direction can meet the application requirements. For an event of average urgency, another vertical strip of SAR is added to the front of the base. For a high-urgency event, adding another 1–2 SARs can meet the needs of applications.

### 5.2. Performance Analysis

This section provides the performance results of our proposed ACCDS scheme.

(1) Analysis of communications costs.

**Theorem** **1.** *In the ACCDS scheme, the ratio of the cost from the disseminated actuator location to the cost of flooding routing is shown in Equation (9):*

(9) $$\theta =2\left(\epsilon_{t}+\epsilon_{r}\right)/\left(\sqrt{ℳ}\left(\epsilon_{t}+8\epsilon_{r}\right)\right)$$

**Proof.**  The communication cost of flooding routing algorithm first can be calculated. In flooding routing, each node broadcasts at least once. Only the GH can receive and send the broadcast information. Eight neighbor GHs can receive data when the information can be broadcast by the GH. The energy consumption for receiving a packet is $\epsilon_{r}$, and the energy consumption for sending a packet is $\epsilon_{t}$. Thus, the energy consumption for broadcasting information once is $\epsilon_{t}+8\epsilon_{r}$. The total number of GHs is ℳ; therefore, the energy consumption is $ℳ\epsilon_{t}+8\epsilon_{r}$. There are N actuators; the total energy consumption is $Nℳ\left(\epsilon_{t}+8\epsilon_{r}\right)$. In the ACCDS scheme, the cost for launching the location dissemination of actuators is Equation (6). The number of actuators is N; the energy consumption is $2N\sqrt{ℳ}\left(\epsilon_{t}+\epsilon_{r}\right)$. Thus, Equation (9) can be proven. ☐

The cost of actuator location dissemination in the ACCDS scheme is considerably lower than the cost of the flooding routing scheme. In the flooding routing scheme, all nodes broadcast information over the entire network and receive messages. In the ACCDS scheme, however, the information is unicast broadcast; a small portion of the node can participate in the broadcast information, rendering the cost small.

**Theorem** **2.** *In the ACCDS scheme, the cost* $c_{3}$ *for identifying the actuators of an event is shown in Equation (10):*

(10) $$c_{3}=N\sqrt{ℳ}\bar{X}\left(1+aℰ\right)\left(\epsilon_{t}+\epsilon_{r}\right)$$

*where* a *is the average number of SARs for updating actuators and* ℰ *is a decimal in the range of (0, 1).*

**Proof.**  In the ACCDS scheme, the average number for sending SAR of actuators is $\bar{X}$. The longest path for each SAR is $\sqrt{2ℳ}$. The number of branch routes for launching SAR is a, and the average length of each branch is $ℰ\sqrt{2ℳ}$. The total energy consumption is $\bar{X}\left(\sqrt{2ℳ}+aℰ\sqrt{2ℳ}\right)\left(\epsilon_{t}+\epsilon_{r}\right)$. There are N actuators, and the total energy consumption is $N\bar{X}\left(\sqrt{2ℳ}+aℰ\sqrt{2ℳ}\right)\left(\epsilon_{t}+\epsilon_{r}\right)$. Thus, Equation (10) can be proven. ☐

**Theorem** **3.** *If the cycle of the flooding routing scheme is* Τ*, the average speed of the actuators’ movement is* 𝓋*, and the number of sent messages regarding an event in* Τ *times is* b*. Thus, the ratio of the cost of message routing in the ACCDS scheme and the cost of flooding routing is demonstrated in Equation (11):*

(11) $$\vartheta =\left(D_{b}+D_{dir}/\left(2ℊ\right)\right)/\left(D_{b}+D_{e}/2\right)$$

**Proof.**  In the flooding routing scheme, the movement distance of the actuators in the time *T* is 𝓋Τ. For event e, considering that its initial arrival actuator distance is $D_{b}$, the movement distance at the end of the cycle is $D_{e}=𝓋Τ$. Thus, the average communication length is $D_{b}+D_{e}/2$. The number for the sending packet is ℊ.

In the ACCDS scheme, when encountering Equation (12), shortcutting II routing can be launched, where $D_{dir}$ is the straight line distance from event to actuator and $D_{new}$ is the movement distance of the actuator:

(12) $$ℊ\left(D_{b}+D_{new}-D_{dir}\right)=D_{dir}$$

When the movement distance of the actuator is equal to $D_{new}$ in Equation (13), the straight-line routing can be used in this paper (best choice):

(13) $$D_{new}=D_{dir}/ℊ+D_{dir}-D_{b}$$

In the beginning of the cycle, it can be assumed that $D_{dir}=D_{b}$. Thus, $D_{new}=D_{dir}/ℊ$ can be achieved. In the ACCDS scheme, the average movement length of an event is $D_{b}+D_{new}/2=D_{b}+D_{dir}/\left(2ℊ\right)$. ☐

(2) Analysis of delay.

**Theorem** **4.** *In the ACCDS scheme, for urgent event* e*, the ratio of the length of the passed path, which is observed to be the length of the optimal path, is lower than* $\sqrt{2}$.

**Proof.**  In the ACCDS scheme, for urgent event e, the number of sent SARs is X > N. One of those SARs is sent in a horizontal direction (or a vertical path). A SAR must encounter the location disseminated routing path of the actuator. Thus, the route that locates the actuator must pass the three edges of the triangle although the distance from the event to the actuator is equal to the longest edge of the triangle (see triangle comprising the three edges of $e_{2}IA_{2}$ in Figure 4). ☐

## 6. Experimental Results and Analysis

OMNET++ was used for experimental verification [37]. Without a loss of generality, there are 1000 sensor nodes randomly distributed in a 500 × 500 m^2^ area, and the wireless transmission range of each node is 50 m. The ACCDS scheme compares two schemes: (1) in the CODE scheme, an event receives the location of all actuators by diffusing the location information at the beginning. The GH of a cell located by an actuator can track the movement path of the actuator when the actuator moves to another position. When an event occurs after a period of time, the event message can be sent to the original location of the actuators and then sent to actuators along the tracking path; (2) in the location information diffusion (LID) scheme, when an actuator moves and an event occurs after a period of time, the location information can be diffused throughout the network to obtain the last location of the actuator.

### 6.1. Experimental Results of the Hop Number from Source to Actuator

**Figure 6:**
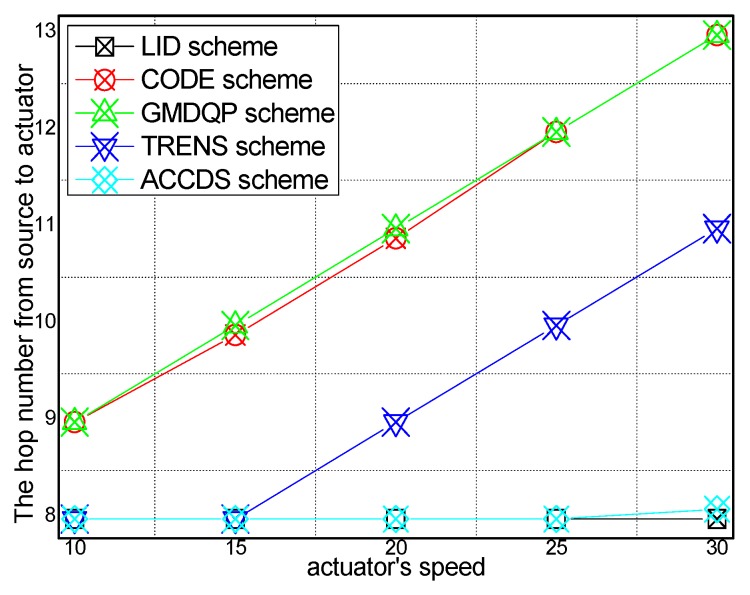
The hop number from source to actuator with different actuator speeds.

**Figure 7:**
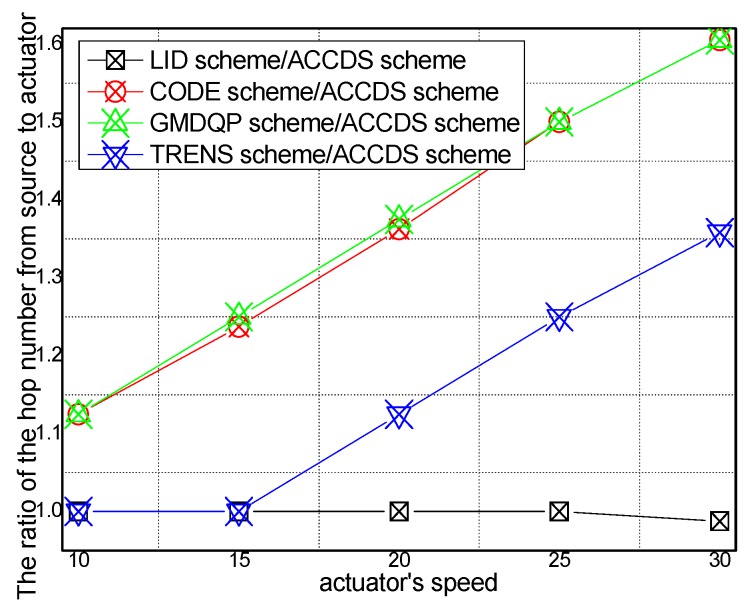
The ratio of the hop number from source to actuator with different actuator speeds.

Figure 6 and Figure 7, respectively, present the hop number for determining the location of an actuator with different actuator speeds in different schemes and the ratio of the hop number for determining the location of an actuator with different actuator speeds in the ACCDS scheme compared with other schemes. Figure 6 demonstrates that the hop number for determining the location of an actuator in the ACCDS scheme is greater than the hop number of the LID scheme; however, the hop number for determining the location of an actuator in the ACCDS scheme is smaller than the number of the TRENS scheme. The hop number for determining the location of actuation in the ACCDS scheme and the LID scheme increases with the speed of the actuator. In fact, the hop count for creating routing from an event to actuators is the primary factor affecting delay; thus, the hop count is used to measure the delay. Figure 7 demonstrates that the hop number for determining the location of an actuator in the TRENS scheme is 1–1.35802 times than that of ACCDS scheme. In the ACCDS scheme, when an event occurs, the source node of the event sends appropriate SAR to obtain the location of the actuator, allowing the source node of the event to send a message to the actuator quickly. 

**Figure 8:**
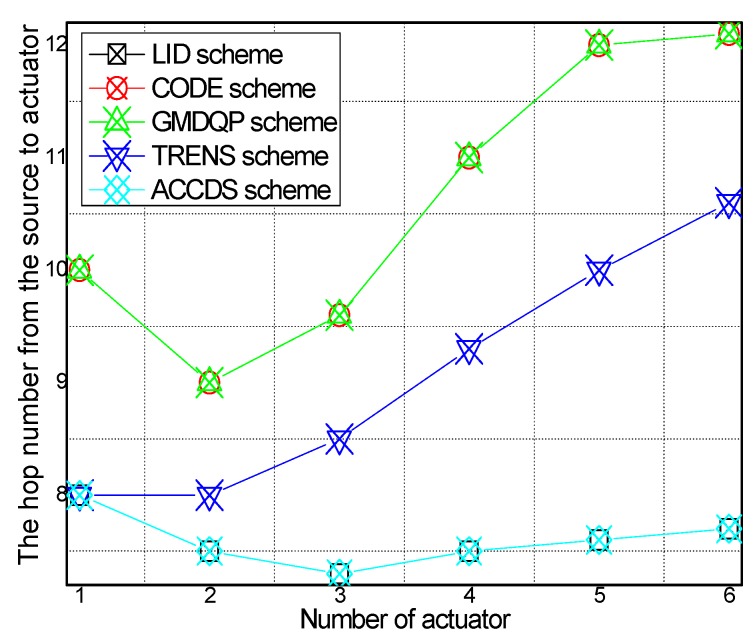
The hop number from source to actuator with different numbers of actuators.

**Figure 9:**
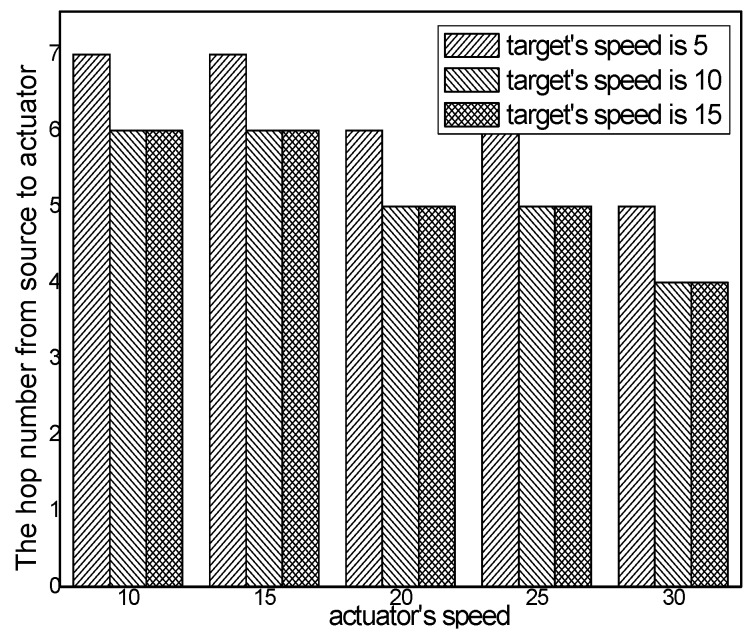
The hop number from source to actuator with different actuator speeds.

The hop number from source to actuator with different numbers of actuators in different schemes and the hop number from source to actuator in the ACCDS scheme with the target and the actuators moving at different speeds are presented in Figure 8 and Figure 9, respectively. Several conclusions can be drawn from Figure 8: The hop number from source to actuator in the ACCDS scheme is smaller than in the other schemes (CODE scheme, GMQDP scheme and TRENS scheme). The reason is the same as before. Figure 9 demonstrates that the hop number from source to actuator decreases with the speed of the actuators, and the hop number from source to actuator decreases as the target’s speed increases.

**Figure 10:**
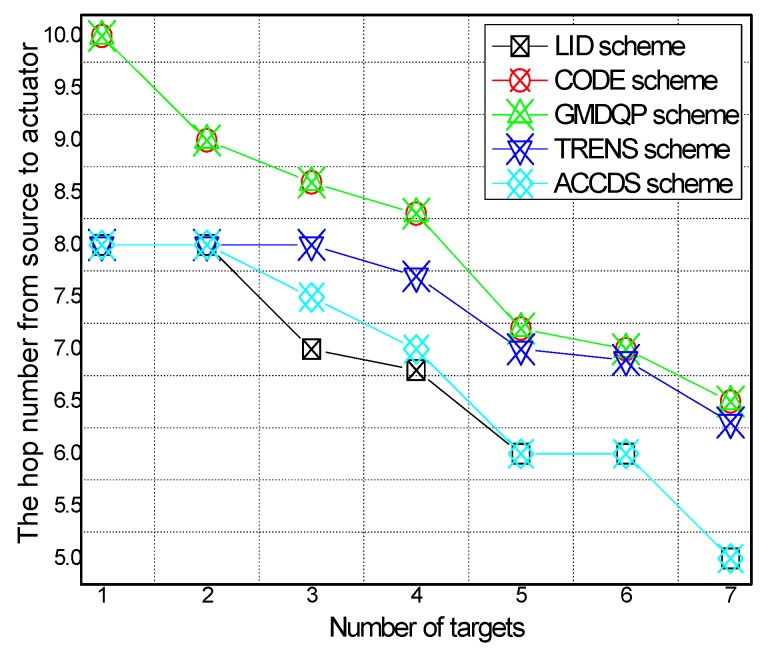
The hop number from source to actuator with different numbers of targets.

**Figure 11:**
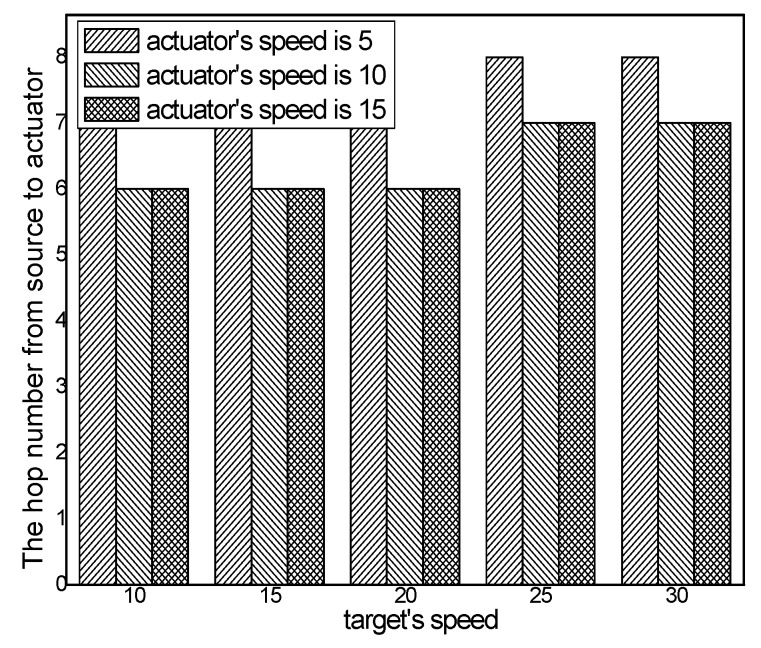
The hop number from source to actuator in the ACCDS scheme with different actuator speeds and different target speeds.

The hop number from source to actuator with different numbers of targets in different schemes is presented in Figure 10, and the hop number from source to actuator in the ACCDS scheme with different actuator speeds with targets moving at different speeds is presented in Figure 11. Figure 10 indicates that the hop number from source to actuator in the ACCDS scheme is greater than in the LID scheme. The reason is that in the LID scheme, to obtain the location of the actuator, the source node of the event diffuses information over the entire network; thus, the location can be obtained quickly once the location of the actuator is determined. However, the energy consumption is considerably greater than for the ACCDS scheme. Although the location of the actuator can be obtained quickly, a huge amount of energy is consumed. The hop number from source to actuator in ACCDS scheme is smaller than in the TRENS scheme. The reason is that the TRENS scheme sends shortcutting packet to build shortcutting path when the actuator moves. The ACCDS scheme sends SAR to determine the location of actuators directly. Thus the hop number from source to actuator in the ACCDS scheme is smaller than in the TRENS scheme. Figure 11 shows that the hop number from source to actuator increases as the actuator’s speed increases, and the hop number for from source to actuator decreases with the speed of the targets.

### 6.2. Experimental Results of Transmission Delay

Though the data transmission in MAC layer and link layer are simplified, the MAC, queuing, propagation are considered in this paper. The transmission delay is refer to the time from the time that source node send message to the time that the actuator receive message.

**Figure 12:**
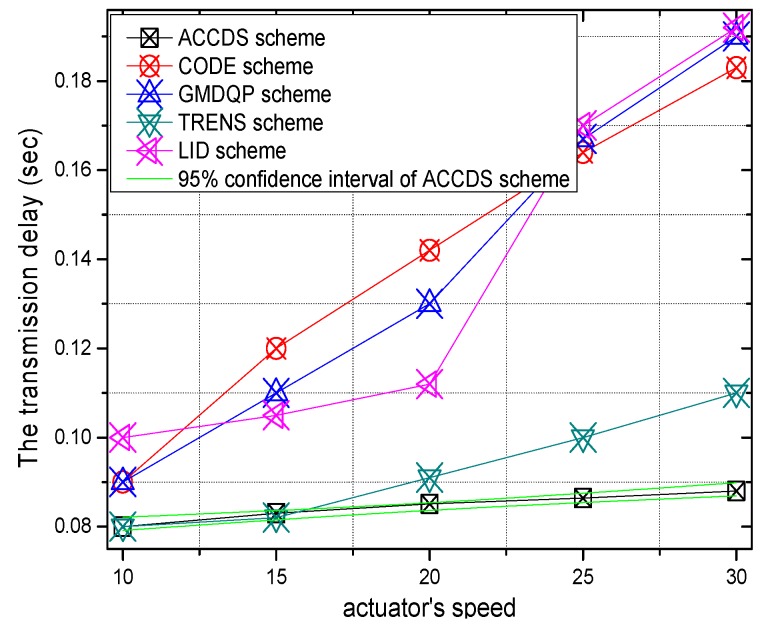
The hop number from source to actuator with different actuator speeds.

**Figure 13:**
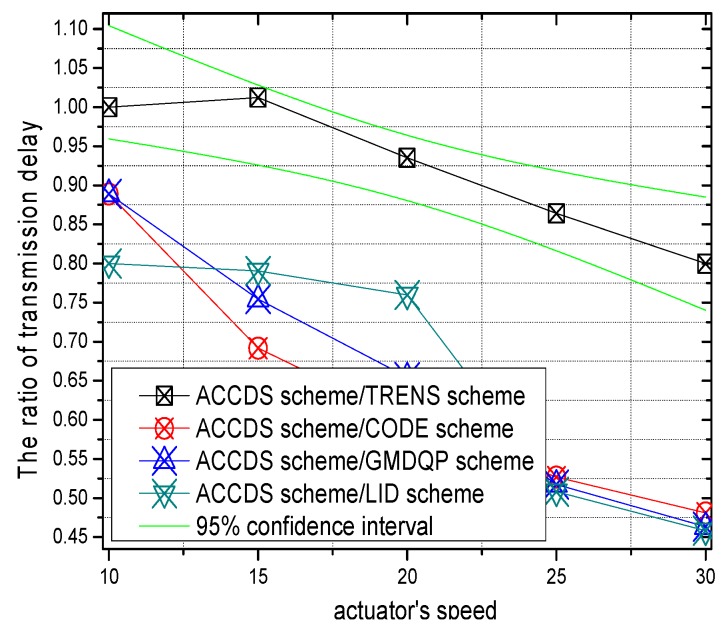
The ratio of the hop number from source to actuator with different actuator speeds.

The transmission delays with different actuator speeds in different schemes and the ratio of the transmission delay with different actuator speeds in the ACCDS scheme compared with other schemes are presented in Figure 12 and Figure 13, respectively. Figure 12 demonstrates that the transmission delay in the ACCDS scheme is smaller than in the other schemes (CODE scheme, GMDQP scheme and TRENS scheme). However, the transmission delay increases with the speed of the actuator. Figure 13 indicates that the transmission delay in the ACCDS scheme is 0.46316–0.88889 times greater than for the TRENS scheme. The reason is that when an event occurs in the ACCDS scheme, the source node of the event sends the appropriate SAR to obtain the location of the actuator, and then the sensing information can be sent to the actuator along the nearest path according to the obtained location of actuator. For the TRENS scheme, however, when an event occurs, the information must be sent along the shortcutting path of the TRENS scheme, rendering longer time for completing the shortcutting path in TRENS scheme. Consequently, the ACCDS scheme performs better.

**Figure 14:**
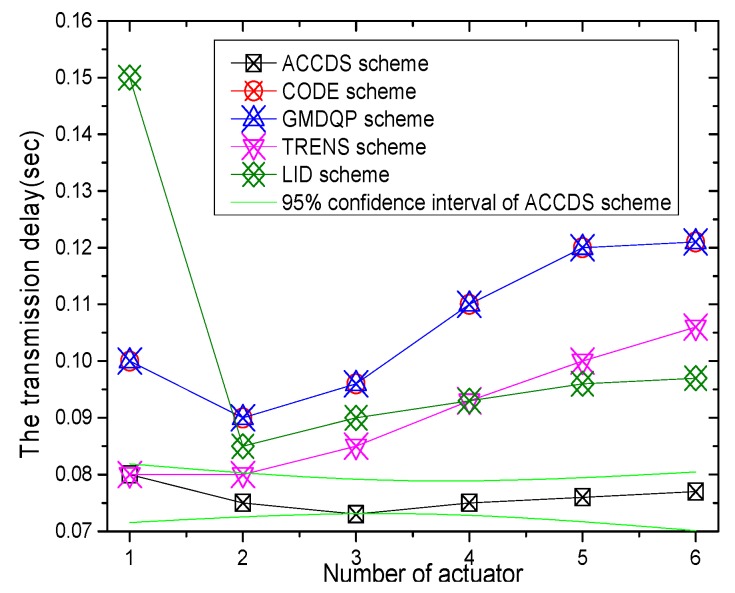
The hop number from source to actuator with different numbers of actuators.

**Figure 15:**
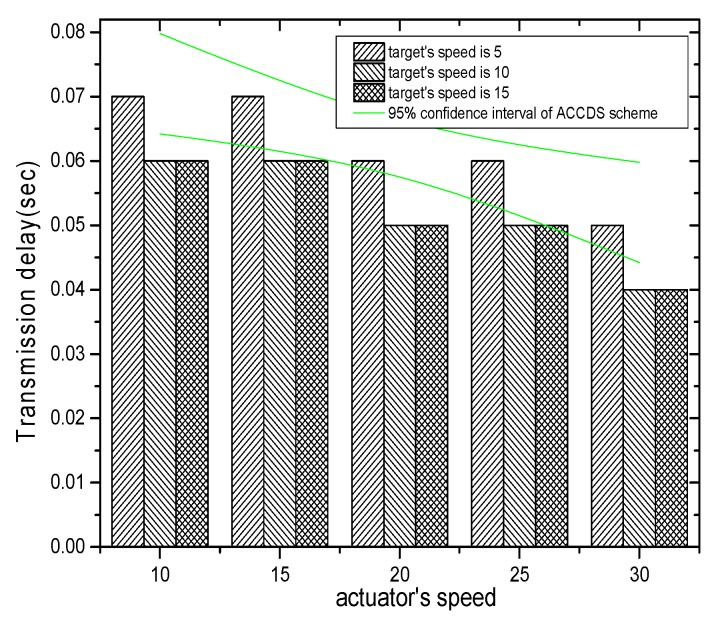
The hop number from source to actuator with different actuator speeds.

The transmission delay with different numbers of actuators in different schemes and the transmission delay in the ACCDS scheme with different target speeds and different actuator speeds are presented in Figure 14 and Figure 15, respectively. Figure 14 indicates that the transmission delay to actuator changes less as the number of actuators increases; however, the transmission delay in the ACCDS scheme is smaller than for the TRENS scheme. The reason is that the TRENS scheme need time to build the shortcutting path, though the TRENS scheme uses a shortcutting path, the routing path is still longer than in the ACCDS scheme. In the ACCDS scheme, however, the source GH can also send information directly once the location of the actuator is determined. Figure 15 demonstrates that the transmission delay in the ACCDS scheme increases with the speed of the actuators.

**Figure 16:**
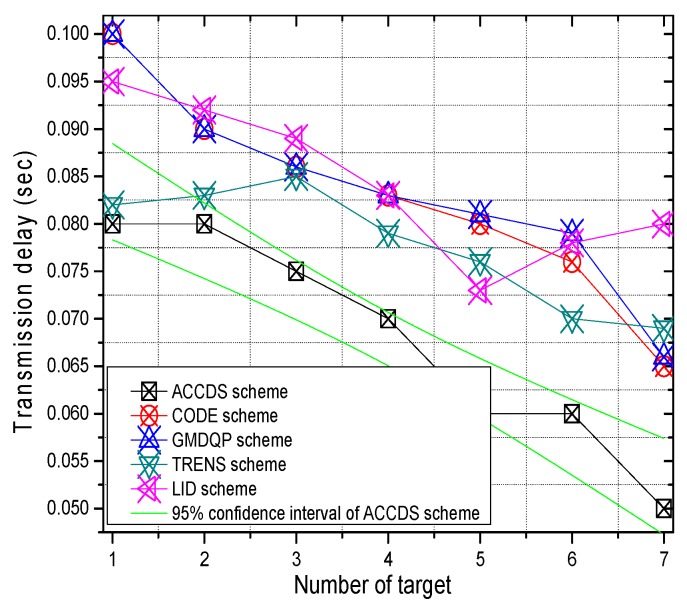
The hop number from source to actuator with different numbers of targets.

**Figure 17:**
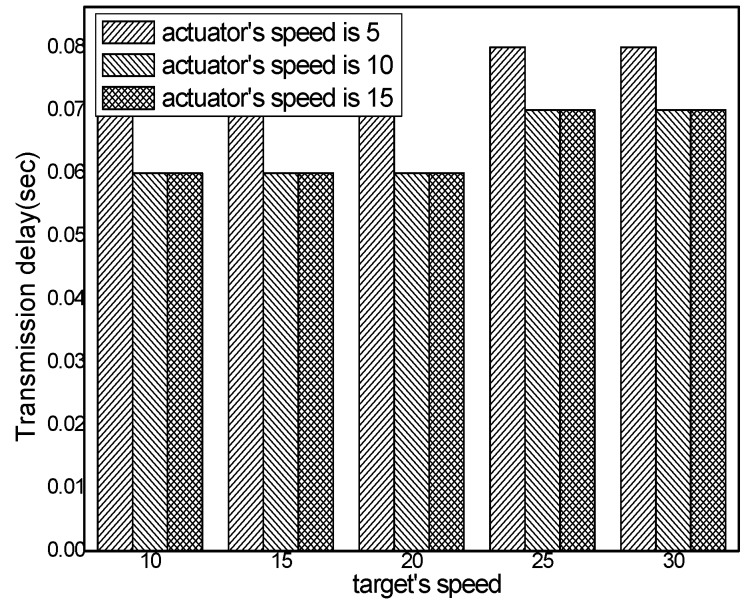
The hop number from source to actuator in the ACCDS scheme with different actuator speeds and different target speeds.

The transmission delay with different numbers of targets in different schemes and the transmission delay in the ACCDS scheme with different actuator speeds and different target speeds are presented in Figure 16 and Figure 17, respectively. Figure 16 indicates that the change in the transmission delay decreases as the number of actuators increases, and the transmission delay in ACCDS scheme is smaller than for the TRENS scheme. 

Figure 17 demonstrates that the transmission delay in the ACCDS scheme declines with the actuator’s speed.

### 6.3. Experimental Results of Energy Consumption

**Figure 18:**
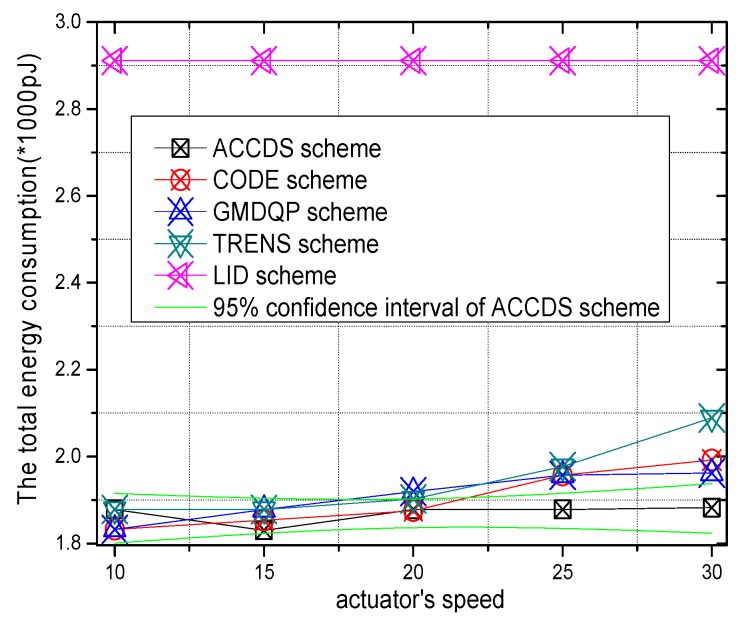
The total energy consumption with different actuator speeds.

**Figure 19:**
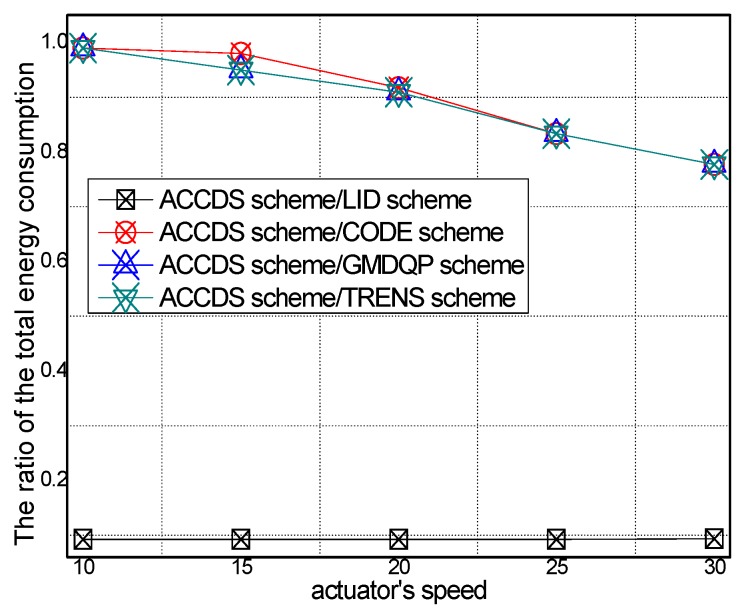
The ratio of total energy consumption in the ACCDS scheme to other schemes.

The logarithmic scale of total energy consumption with different actuator speeds in different schemes and the ratio of total energy consumption in the ACCDS scheme to other schemes with different actuator speeds are presented in Figure 18 and Figure 19, respectively. Figure 18 demonstrates that the total energy consumption in the ACCDS and other schemes (CODE scheme, GMDQP scheme and TRENS scheme) is smaller than for the LID scheme. Figure 19 demonstrates that the total energy consumption in the ACCDS scheme is only 0.09259–0.09352 times greater than for the LID scheme, and the total energy consumption in the ACCDS scheme is 0.77692–0.95 times greater than for the TRENS scheme. In TRENS scheme, the routing path is longer than for the ACCDS scheme, and the shortcutting packet is sent to build the shortcutting path, rendering energy consumption higher. In the ACCDS scheme, however, the source node of the event only sends appropriate SAR to determine the location of actuation. Thus, the energy consumption of the LID scheme is the greatest.

**Figure 20:**
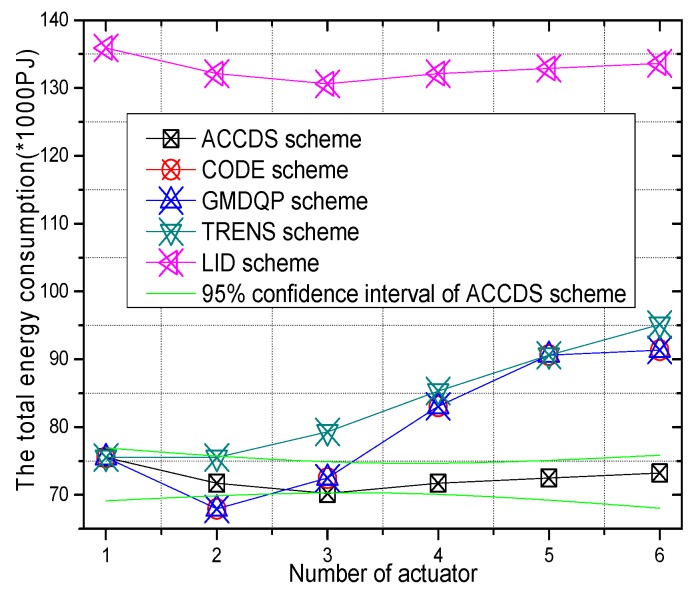
The total energy consumption with different numbers of actuators.

**Figure 21:**
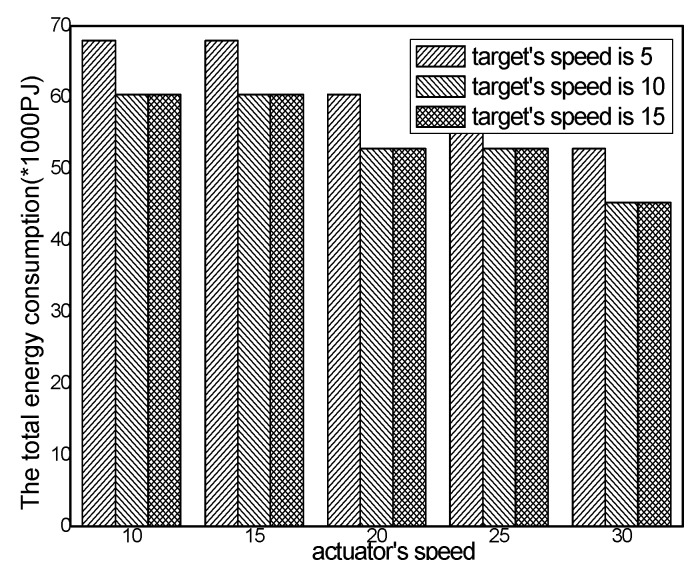
The total energy consumption with different actuator speeds in the ACCDS with different target speeds.

The total energy consumption with different numbers of actuators in different schemes and the total energy consumption with different actuator speeds in the ACCDS with different target speeds are presented in Figure 20 and Figure 21. Figure 20 demonstrates that the total energy consumption in the ACCDS and other schemes (CODE scheme, GMDQP scheme and TRENS scheme) is smaller than in the LID scheme. Figure 21 indicates that the total energy consumption in the ACCDS scheme decreases with the increase in actuator speed.

**Figure 22:**
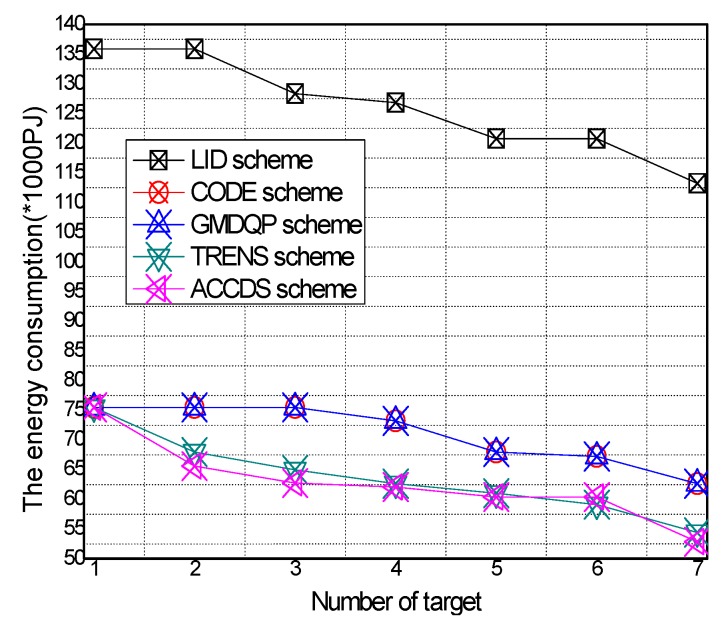
Total energy consumption with different numbers of targets.

**Figure 23:**
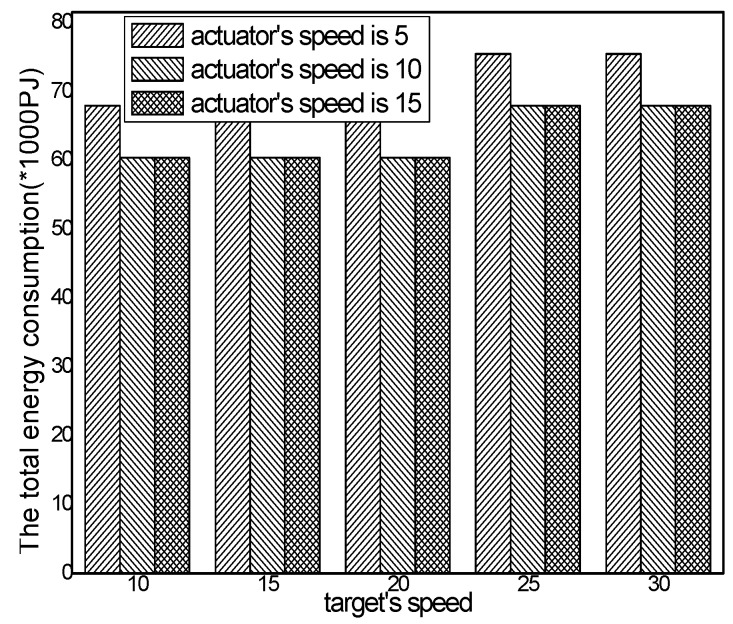
Total energy consumption with different target speeds in the ACCDS with different actuator speeds.

The total energy consumption with different numbers of targets in different schemes and the total energy consumption with different target speeds in the ACCDS with different actuator speeds are presented in Figure 22 and Figure 23. Figure 22 demonstrates that the total energy consumption in the ACCDS and other schemes (CODE scheme, GMDQP scheme and TRENS scheme) is smaller than in the LID scheme. Figure 23 indicates that the total energy consumption in the ACCDS scheme increases with the increase in target speed. Consequently, the ACCDS scheme performs better.

### 6.4. Network Lifetime

**Figure 24:**
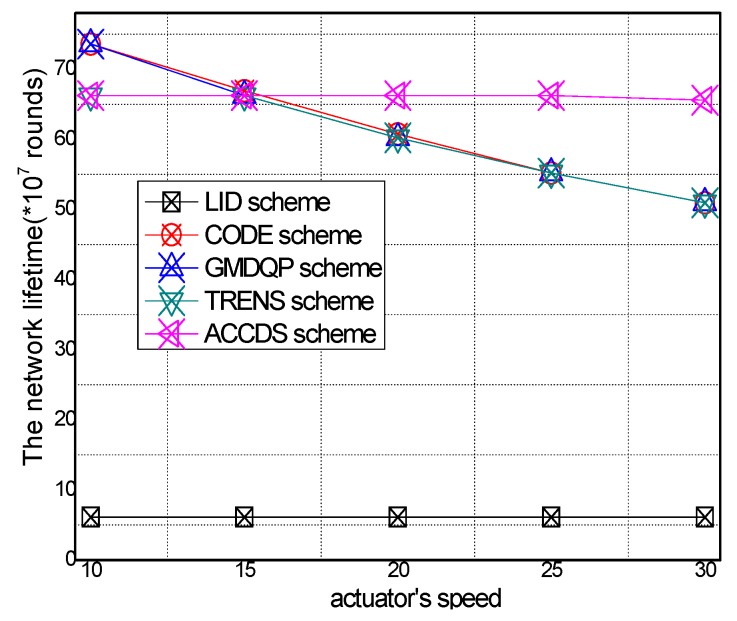
Total energy consumption with different numbers of targets.

**Figure 25:**
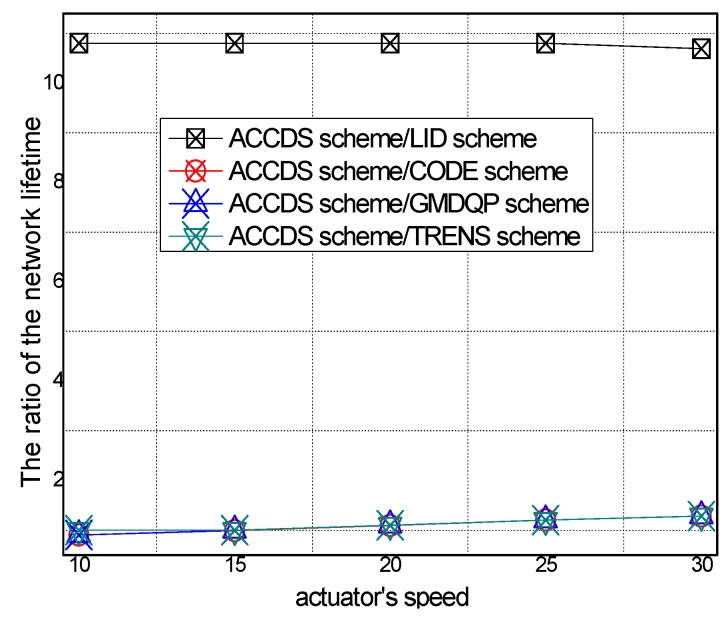
Total energy consumption with different target speeds in the ACCDS with different actuator speeds.

The network lifetime in different schemes and the ratio of T network lifetime in the ACCDS scheme to other schemes with different actuator speeds are presented in Figure 24 and Figure 25, respectively. Figure 24 demonstrates that the network lifetime in the ACCDS and other schemes (CODE scheme, GMDQP scheme and TRENS scheme) is higher than for the LID scheme. Figure 25 demonstrates that the total energy consumption in the ACCDS scheme is reduced up to about 0.28713 compared with the TRENS scheme, In TRENS scheme, the routing path is longer than for the ACCDS scheme, and the shortcutting packet is sent to build the shortcutting path, rendering network lifetime lower.

**Figure 26:**
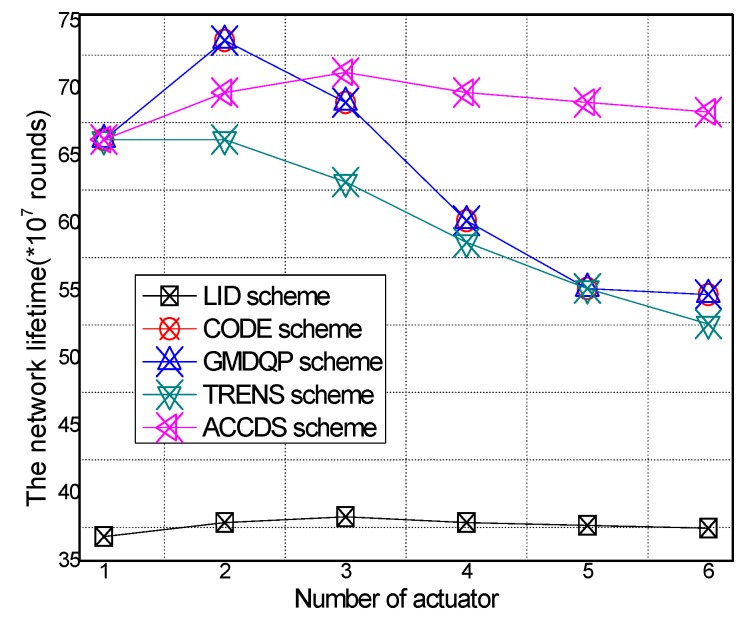
Total energy consumption with different numbers of targets.

**Figure 27:**
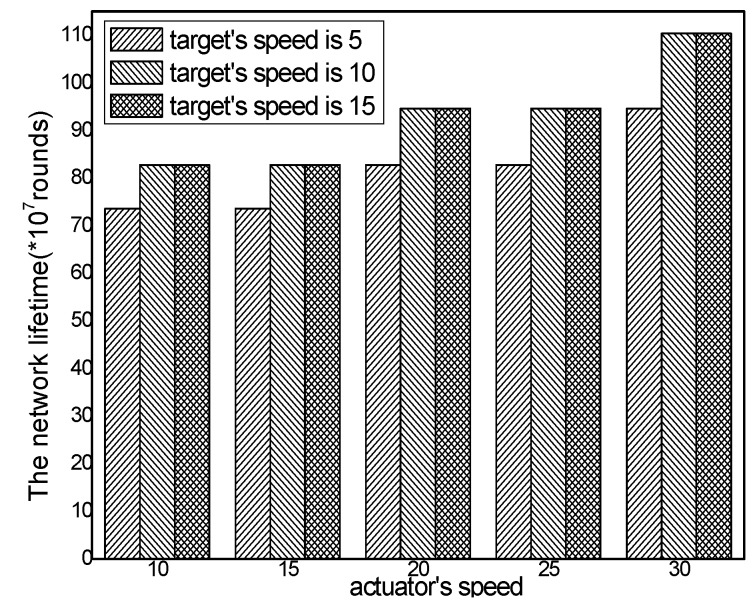
Total energy consumption with different target speeds in the ACCDS with different actuator speeds.

The network lifetime with different numbers of actuators in different schemes and the total energy consumption with different actuator speeds in the ACCDS with different target speeds are presented in Figure 26 and Figure 27. Figure 26 demonstrates that the network lifetime in the ACCDS and other schemes (CODE scheme, GMDQP scheme and TRENS scheme) is higher than in the LID scheme. Figure 27 indicates that the network lifetime in the ACCDS scheme increases with the increase in actuator speed.

**Figure 28:**
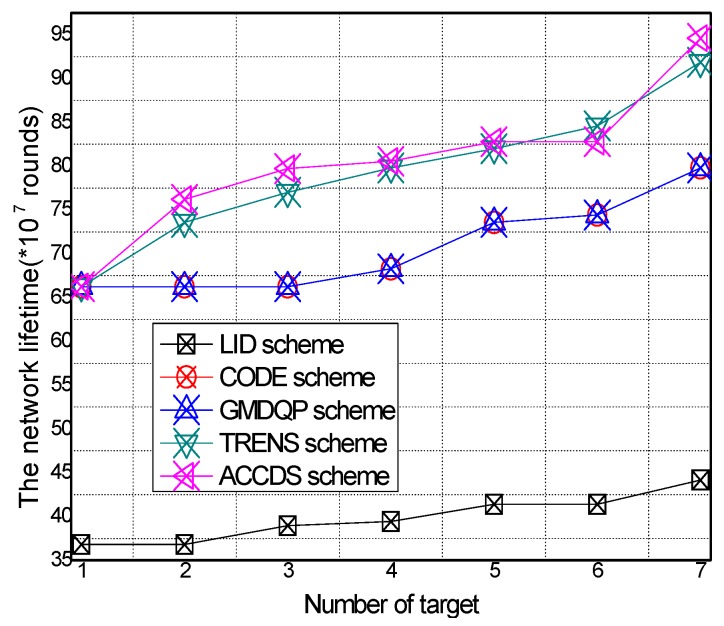
Total energy consumption with different numbers of targets.

**Figure 29:**
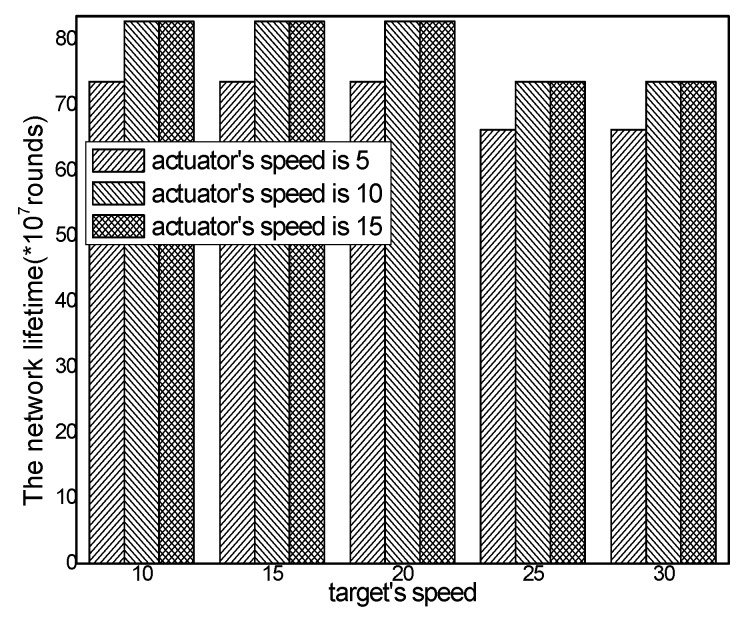
Total energy consumption with different target speeds in the ACCDS with different actuator speeds.

The network lifetime with different numbers of targets in different schemes and the total energy consumption with different target speeds in the ACCDS with different actuator speeds are presented in Figure 28 and Figure 29. 

Figure 28 demonstrates that the network lifetime in the ACCDS and other schemes (CODE scheme, GMDQP scheme and TRENS scheme) is higher than that in the LID scheme. Figure 29 indicates that the network lifetime in the ACCDS scheme decreases with the increase in target speed.

## 7. Conclusions and Future Work

Events and actuators can be deployed in WSANs, the event and the actuators in WSANs can both move and connect with various QoS requirements of an event to cause greater challenges for this study. This paper proposes an adaptive communication control based on a differentiated delay (ACCDS) scheme to provide different QoS for different applications. The following pivotal methods were adopted to achieve the goal of the ACCDS scheme: (1) actuators adopt the limited position diffusion protocol based on the minimum cost principle, thus changing the shorting of higher costs of flooding routing and improve the network lifetime; (2) the event adaptively sends different numbers of SARs according to the different degrees of sensitivity to delay so that the tradeoff between delay and communication costs can be optimized; thus improve the network lifetime; (3) actuators initiate shortcutting routing based on network status and further improve network performance. The theoretical and experimental results demonstrate the effectiveness of the ACCDS scheme.

## Figures and Tables

**Table 1:** Network parameters.

Parameter	Value
Threshold distance (*d*_0_) (m)	87
Sensing range *r_s_* (m)	15
*E_elec_* (nJ/bit)	50
*e_fs_* (pJ/bit/m^2^)	10
*e_amp_* (pJ/bit/m^4^)	0.0013
Initial energy (J)	0.5
